# Enhanced production of polyhydroxybutyrate by multiple dividing *E. coli*

**DOI:** 10.1186/s12934-016-0531-6

**Published:** 2016-07-27

**Authors:** Hong Wu, Zhongyun Fan, Xiaoran Jiang, Jinchun Chen, Guo-Qiang Chen

**Affiliations:** 1 School of Life Sciences, Tsinghua University, Beijing, 100084 China; 2 Center for Synthetic and Systems Biology, Tsinghua University, Beijing, 100084 China; 3 Tsinghua-Peking Center for Life Sciences, Tsinghua University, Beijing, 100084 China; 4 Center for Nano and Micro Mechanics, MOE, Tsinghua University, Beijing, 100084 China; 5 MOE Key Lab of Industrial Biocatalysis, Dept Chemical Engineering, Tsinghua University, Beijing, 100084 China

**Keywords:** Cell fission, Growth pattern, PHB, *Escherichia coli*, Cell morphology, Inclusion body, Synthetic biology

## Abstract

**Background:**

Most bacteria are grown in a binary fission way meaning a bacterial cell is equally divided into two. Polyhydroxyalkanoates (PHA) can be accumulated as inclusion bodies by bacteria. The cell division way and morphology have been shown to play an important role in regulating the bacterial growth and PHA storages.

**Results:**

The common growth pattern of *Escherichia coli* was changed to multiple fission patterns by deleting fission related genes *minC* and *minD* together, allowing the formation of multiple fission rings (Z-rings) in several positions of an elongated cell, thus a bacterial cell was observed to be divided into more than two daughter cells at same time. To further improve cell growth and PHA production, some genes related with division process including *ftsQ*, *ftsL*, *ftsW*, *ftsN* and *ftsZ*, together with the cell shape control gene *mreB*, were all overexpressed in *E. coli* JM109 *∆minCD*. The changing pattern of *E. coli* cell growth and morphology resulted in more cell dry weights (CDW) and more than 80 % polyhydroxybutyrate (PHB) accumulation increases compared to its binary fission control grown under the same conditions.

**Conclusions:**

This study clearly demonstrated that combined over-expression genes *ftsQ*, *ftsW*, *ftsN*, *ftsL* and *ftsZ* together with shape control gene *mreB* in multiple division bacterial *E. coli* JM109 *∆minCD* benefited PHA accumulation. Our study provides useful information on increasing the yield of PHA by changing the cell division pattern and cell morphology of *E. coli*.

**Electronic supplementary material:**

The online version of this article (doi:10.1186/s12934-016-0531-6) contains supplementary material, which is available to authorized users.

## Background

In *Escherichia coli*, cell division is regulated strictly to ensure that daughter cells contain proper cellular components [1]. Most bacteria are divided via binary fission, allowing a parent cell to split in two [2]. During this process, several genes and their products are important, such as the tubulin-like protein FtsZ [3], and MinC, MinD and MinE that regulate the formations of division sites [4, 5]. The MinC and MinD are inhibitors able to block the formation of the FtsZ ring at all sites, while MinE relieves the division block at the mid-cell site, resulting in binary fission [4, 6]. In the absence of MinC and MinD, the divisions appear to form at all division sites since FtsZ rings can be generated at both polar and medial positions [7, 8]. On the other hand, deletion of the ‘min’ system results in the production of some mini-cells, products of divisions near the *E. coli* cell poles [9]. The cell division pattern could be changed by disrupting the ‘*min’* system, allowing the common binary fission be changed to multiple fissions [10].

To change the replication process of a bacterial cell, the formation of FtsZ ring and proper septation should be manipulated [6]. There are at least ten genes that have been shown to be essential for formation of the FtsZ ring and regulation the division process [11, 12]. Among the essential genes, FtsQ and FtsL are two membrane proteins localizing to the cell septum during division process [13, 14], and the location of FtsW is dependent on the prior localization of FtsQ and FtsL [15, 16]. As the last protein acted in cell division, FtsN causes the disassembly of other elements from the division ring [17, 18]. FtsZ interacts with FtsQ, FtsL, FtsW and FtsN in the progression and completion of cytokinesis [12]. FtsZ also plays an important role in the bacterial cell division process as a tubulin-like protein [12, 19, 20].

Polyhydroxyalkanoates (PHA), a family of biodegradable and biocompatible thermal polyesters or bioplastics, are accumulated as inclusion bodies by bacteria under unbalanced growth conditions [21–23]. Polyhydroxybutyrate (PHB) is the model PHA used for many demonstration studies, and it has been developed as environmentally friendly bioplastics with promising applications [24, 25]. High cost of PHA production has been a key limiting factor on its commercial application [26]. Efforts on process optimization, use of cheap carbon sources and pathway engineering were made to cut cost [27–31]. Although the cost of PHA production can be reduced under these efforts, it is still significantly higher compared with the petrochemical plastics such as polyethylene (PE) [24]. Therefore, other methods are needed to reduce the cost of PHA [32, 33].

Since PHA are produced by bacteria as inclusion bodies, cell shapes of the host strain can affect the amount of PHA granules and the quantity of PHA that can be stored [34, 35]. The change of cell division process could produce more daughter cells in various shapes at the same time, possibly leading to more PHA, as was indicated by previous studies that PHA synthesis is also limited by the small cell size, a large cell size with more space can allow more PHA granules to be accumulated. Bacterial peptidoglycan cell wall and the actin-like protein MreB cytoskeleton are major determinants of cell shape in rod-shaped bacteria such as *E. coli* [36–38].

In this study, we aimed to change the cell division pattern and thus cell morphology, and to use the multiple fission cells for possible enhanced PHB accumulation.

## Results

### Changing *E. coli* growth pattern: from binary division to multiple fission

**Fig. 1:**
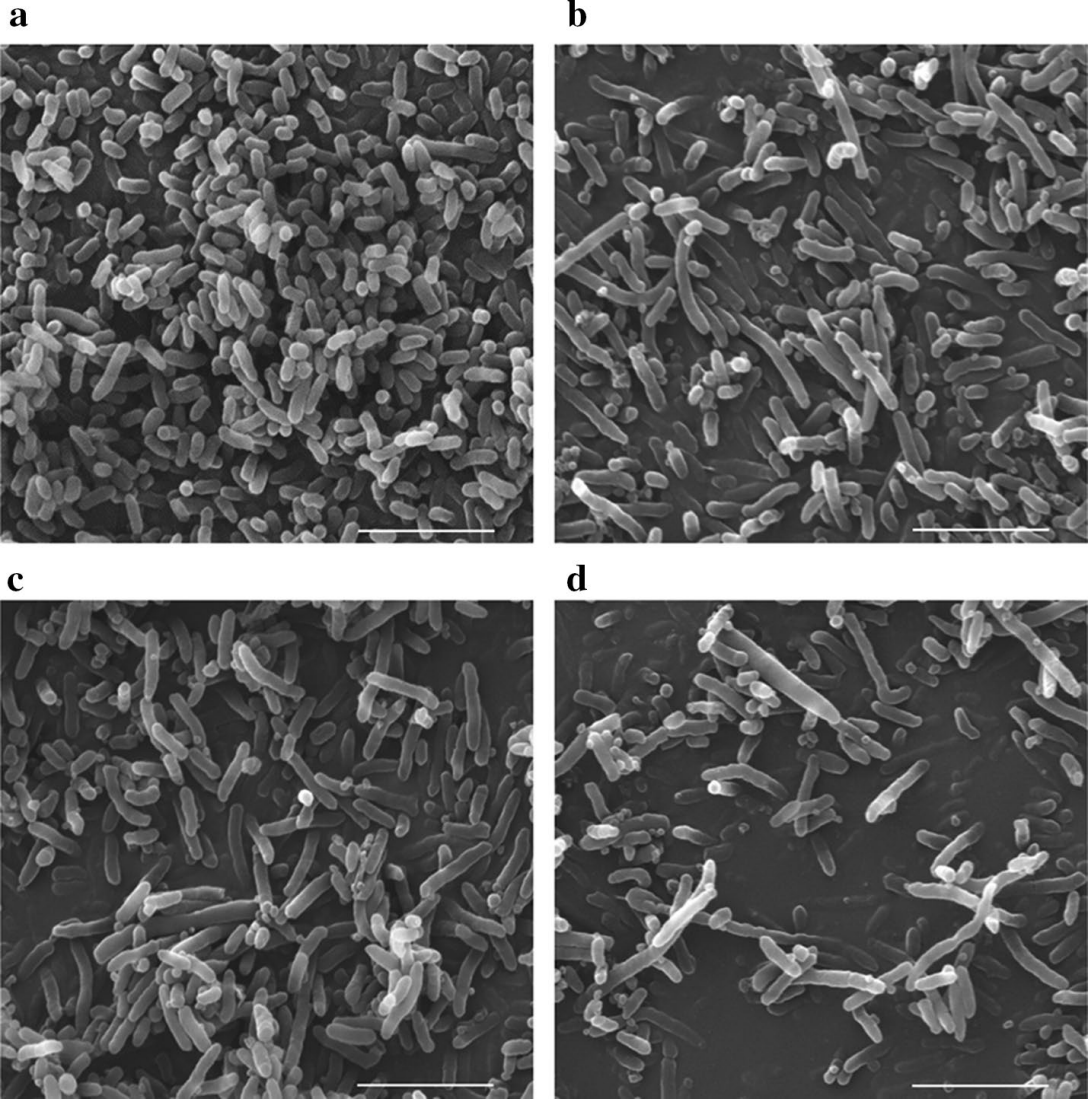
Electron microscopy study on morphology of *E. coli* JM109 deleting *minC* or/and *minD*. **a** *E. coli* JM109. **b** *E. coli* JM109*∆minC*. **c** *E. coli* JM109*∆minD*. **d** *E. coli* JM109*∆minCD*. Cells were all cultivated in LB medium under 37 °C, respectively. *Scale bar* 5 μm

In this study, genes *minC* and *minD* regulating fission ring locations were deleted in *E. coli* JM109 using homologous recombination method. *E. coli* JM109 *∆minCD* became several folds longer than the wild type when cultivated in LB medium (Fig. 1), sizes extended from 1–3 μm for the wild type (Fig. 1a) to around 5 μm for the *E. coli* JM109 *∆minCD* (Fig. 1d), accompanied by some mini-cells attached around the elongated cells. Interestingly, the individual mutant of *minC* and *minD* in *E. coli* JM109, respectively, namely, *E. coli* JM109 *∆minC* (Fig. 1b) and *E. coli* JM109 *∆minD* (Fig. 1c), displayed a similar morphology to *E. coli* JM109 *∆minCD* (Fig. 1d). The reason may be attributed to joint efforts of *minC* and *minD* to decide FtsZ ring formation [7]. Both are essential for the function of ‘*min’* system.

**Fig. 2:**
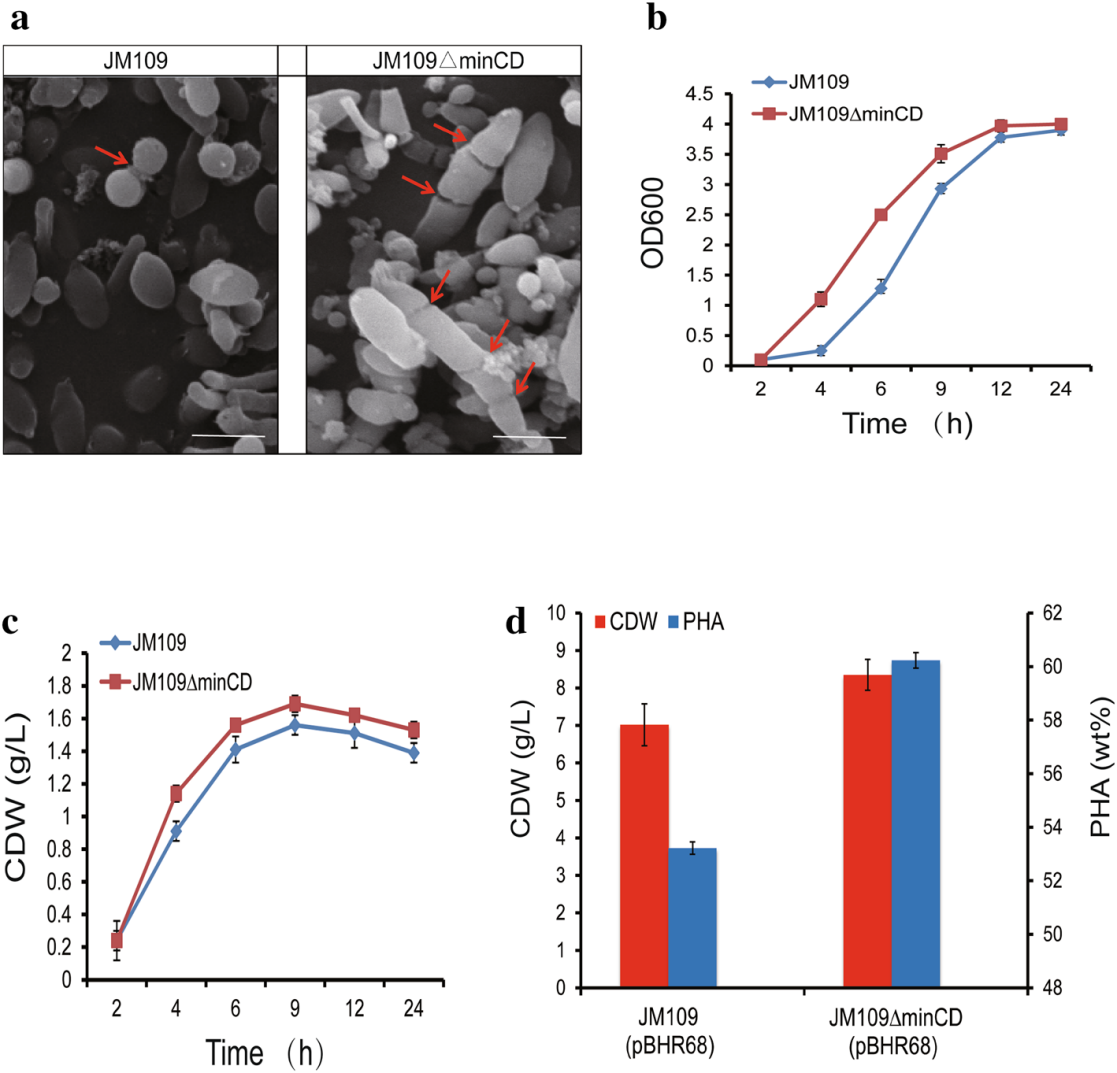
Cell division pattern, morphology and PHA production of *E. coli* JM109*∆minCD*. **a** The division pattern of *E. coli* JM109 *∆minCD* and *E. coli* JM109. *Arrows* the division position of cell. *E. coli* JM109 *∆minCD* had more than two division rings. *Scale bar* 2 μm. The OD_600_ (**b**) and cell dry weight (**c**) of recombinant *E. coli* JM109 *∆minCD*. Cells were cultivated in LB medium under 37 °C for 24 h, respectively. Errors are s.d. (n = 3). **d** The CDW and PHA production by recombinants harboring pBHR68 cultivated in LB medium by addition of 20 g/L glucose at 37 °C for 48 h. *Error bars* are s.d. (n = 3)

In the absences of *minC* and/or *minD*, the FtsZ ring fails to locate to the middle of a cell (Fig. 2a; Additional file 1: Video S1). Under this circumstance, *E. coli* JM109 *∆minCD* changes not only the cell morphology (Fig. 1) but also the way of cell division (Fig. 2a). As multiple FtsZ rings were randomly and simultaneously formed in various positions of an elongated cell of *E. coli* JM109 *∆minCD,* one elongated bacterial cell was broken into more than two daughter cells (Additional file 1: Video S1). For example, a cell of an elongated *E. coli* JM109 *∆minCD* was divided into three daughter cells when two FtsZ rings were formed and located in two different positions of the elongated cell. The size of the daughter cell is dependent on the position where the FtsZ ring is formed. Some mini-cells were observed during the multiple fission process when the FtsZ ring was formed at a polar site where no nucleic acid was available for encapsulating into the cellular space (Fig. 2a). *E. coli* JM109 *∆minCD* changes not only its cell size but also its way of growth.

Growth rate from the binary division to multiple fission was investigated in terms of the OD_600_ and cell dry weight (CDW) of the wild type and mutant grown in LB medium for 24 h, respectively. Even though OD_600_ of *E. coli* JM109 *∆minCD* showed a little bit higher than that of the wild type (Fig. 2b), the cell dry weights indicated only little significant difference between the mutant and wild type (Fig. 2c). OD values were not only related to cell density but also to the cell morphology. Thus, the mutant strain could achieve higher OD value since the cell length was changed. However, the cell dry weight was not enhanced, the reason could be attributed to the mini-cells that could not make a contribution to the final CDW.

When *E. coli* JM109*∆minCD* was used to express PHB synthesis operon *phbCAB* encoded by plasmid pBHR68, it was found that the mutant accumulated slightly more PHB compared with its wild type under the same conditions. When grown in a mineral medium supplemented with yeast extract, the amount of PHB produced by *E. coli* JM109 *∆minCD* had a little increase compared with the wild type *E. coli* JM109. However, the different amounts of yeast extract added into the MM medium (mineral or minimal growth medium) had little effect on the enhancements on cell growth and PHB accumulation (Table 1). When *E. coli* JM109*∆minCD* was cultured in MM medium containing 1 g/L yeast extract, the cell dry weight was 5.12 g/L and PHB contents was 43.08 %, which was higher than the control one and other culture conditions in MM medium. On the other hand, when the mutant was cultivated in LB medium for 48 h, the cell dry weight could reach 8.35 g/L containing 60.23 % PHB, much higher than the control *E. coli* JM109 having only 53.22 % PHB (Fig. 2d). In all cases, LB medium supported better cell growth and PHB synthesis compared with the mineral medium added with yeast extracts (Table 1; Fig. 2d). Since the LB medium was more suitable for *E. coli* JM109*∆minCD*, subsequent study was employed only the LB medium. It was concluded that multiple fission promoted the PHB accumulation compared to the common binary fission way of growth when cultivations were conducted in LB medium.

**Table 1:** Cell growth and PHA accumulation by recombinant *E. coli* grown under different culture conditions

Culture medium	Strain	CDW (g/l)^a^	PHA (wt%)^b^
MM + 0.5 g/L yeast extract	JM109Δ*minCD*	4.96 ± 0.29	39.71 ± 1.72
	JM109	4.41 ± 0.16	36.53 ± 0.24
MM + 1 g/L yeast extract	JM109Δ*minCD*	5.12 ± 0.22	43.08 ± 0.37
	JM109	5.09 ± 0.28	40.81 ± 0.55
MM + 1.5 g/L yeast extract	JM109Δ*minCD*	5.09 ± 0.35	41.26 ± 1.15
	JM109	4.53 ± 0.24	38.19 ± 0.66

^a^Cells were cultivated in mineral medium (MM) added with various amounts of yeast extract containing 20 g/L glucose at 37 °C for 48 h

^b^Data are expressed as the M ± SD, M refers to a mean value and SD standard deviations (n = 3)

### FtsZ, overexpression in *E. coli* JM109*∆minCD* led to multiple dividing cells accumulating more PHB

As an essential cell division protein, FtsZ forms a contractile ring structure (FtsZ ring) at the cell division site, regulation of FtsZ ring assembly controls the timing and the location of cell division [39, 40]. FtsZ ring formed by FtsZ assembly, is a very dynamic process.

**Fig. 3:**
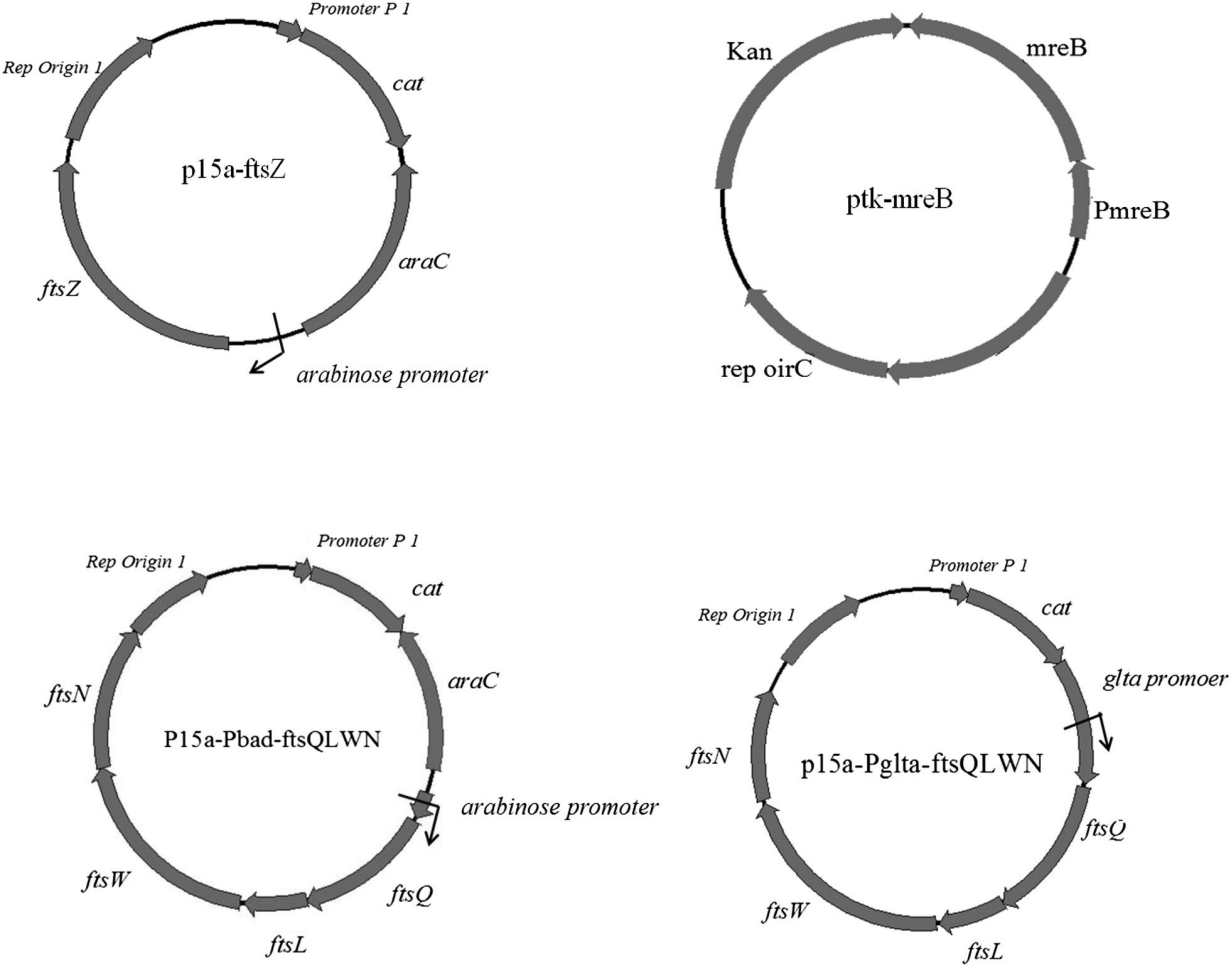
Structures of plasmids used in this study. Plasmid p15a-ftsZ, under an arabinose promoter, was used to control the expression of *ftsZ*; Plasmid ptk-mreB with a *mreB* promoter was constructed; Plasmid p15a-Pbad-ftsQLWN, under the arabinose promoter, was used to control the expression of *ftsQLWN*; Plasmid p15a-Pglta-ftsQLWN, under the *glta* promoter, regulated the expression of *ftsQLWN*

**Fig. 4:**
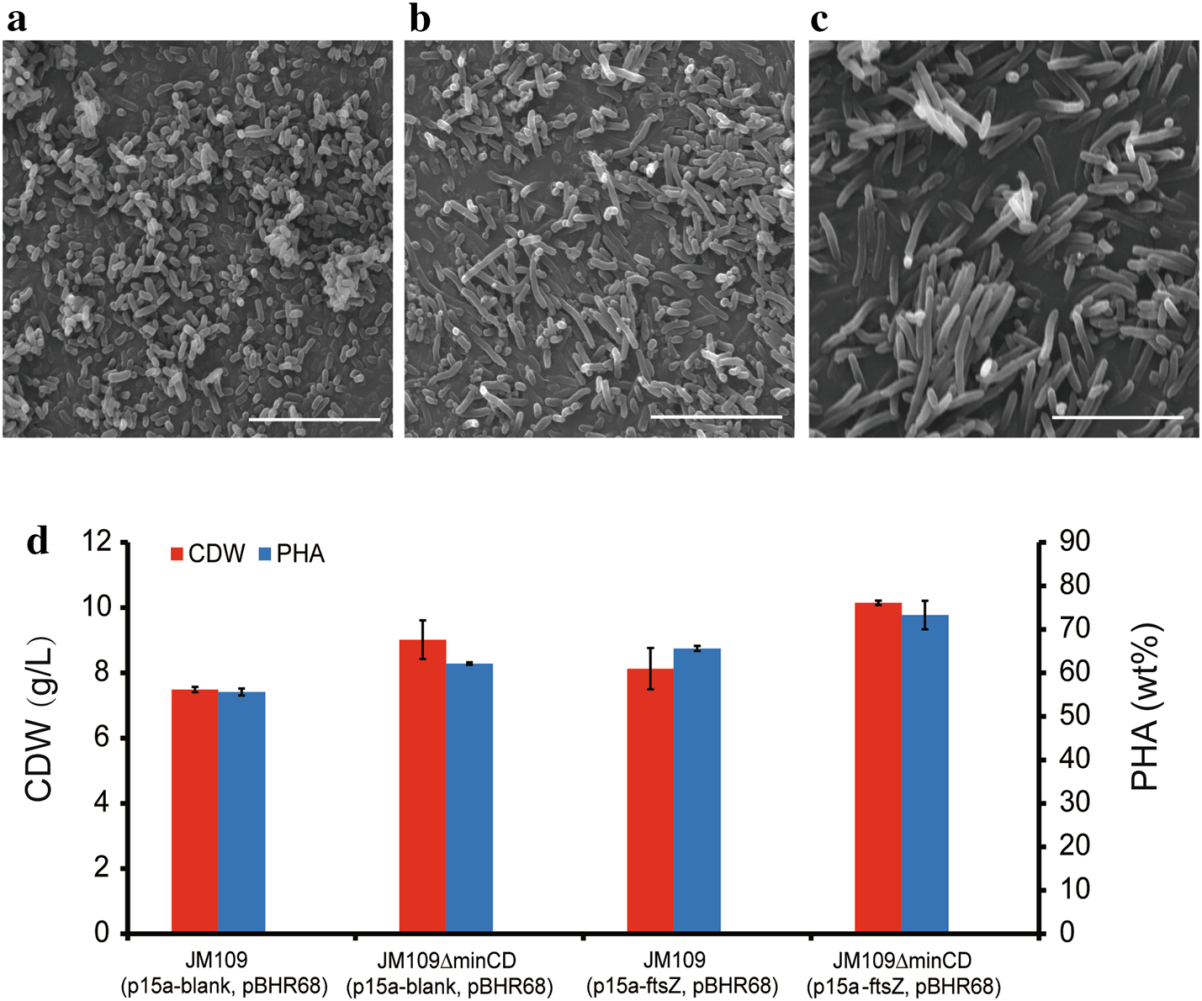
Electron microscopy studies on cell morphology and PHA production of *E. coli* JM109 *∆minCD* overexpressing *ftsZ*. SEM morphology studies on: **a** *E. coli* JM109 (p15a-blank). **b** *E. coli* JM109 *∆minCD* (p15a-blank). **c** *E. coli* JM109 *∆minCD* (p15a-ftsZ). Cells were cultivated in LB medium. *Scale bars*, 10 μm. **d** The cell dry weight and PHA accumulation by *E. coli* JM109 *∆minCD* overexpressing *ftsZ* with pBHR68. Cells were cultivated in LB medium at 37 °C for 48 h, 0.2 % arabinose was added into the medium after 2 h of inoculation, and 20 g/L glucose was added 6 h later. *Error bars* are s.d. (n = 3)

Since location of FtsZ ring was random in *E. coli* JM109*∆minCD*, the elongated cell needed more than two FtsZ rings in order to realize the multiple division. As the correct FtsZ concentration is required for the formation of FtsZ ring, plasmid (p15a-ftsZ) was constructed to regulate the expression level of *ftsZ* under the control of arabinose (Fig. 3). *E. coli* JM109Δ*minCD* harboring plasmid p15a-ftsZ was added with 0.2 % arabinose, *E. coli* JM109Δ*minCD* and *E. coli* JM109 harboring plasmid p15a-blank were used as control. After 2 h inoculation, *E. coli* JM109*∆minCD* (p15a-ftsZ) showed longer cell shape compared with the control groups (Figs. 4a, b, c). The length of *E.coli* JM109*∆minCD* was improved further by overexpression *ftsZ*, and the reason may be that the high expression level of FtsZ disrupted the normal division.

Then, we investigated the influence of *ftsZ* on PHB production. When over-expressing *ftsZ* and PHB synthesis operon *phbCAB* encoded in plasmid pBHR68, *E. coli* JM109*∆minCD* (p15a-ftsZ, pBHR68) grew to 10.15 g/L containing 73.31 % PHB in the cell dry weights, significantly higher than the wild type JM109 (p15a-blank, pBHR68) grew to 7.49 g/L containing 55.62 % PHB (Fig. 4d). Compared with the other control groups, JM109 (p15a-ftsZ, pBHR68) grew to 8.13 g/L with 65.63 % PHB and JM109*∆minCD* (p15a-blank, pBHR68) contained 62.15 % PHB, the ability to produce PHB was improved by overexpressing *ftsZ* in the multiple fission *E. coli* JM109*∆minCD.*

### Over-expressing genes *ftsQ, ftsL, ftsW* and *ftsN* in *E. coli* JM109*∆minCD* improved the PHB contents

To enhance the growth of *E. coli* JM109*∆minCD*, the formation of FtsZ ring should be accelerated to reduce time spent for the cell division (or cell fission) process. Among many essential genes related to the division process, some have showed important functions, including *ftsQ*, *ftsL*, *ftsW* and *ftsN*. FtsZ interacts with these genes encoded proteins to complete cytokinesis [12, 41].

To investigate the influence on growth rate and ability of PHB production by genes *ftsQ*, *ftsL*, *ftsW* and *ftsN*, two types of plasmids were constructed, one was expressed under the control of a constitutive promoter *glta* (p15a-Pglta-ftsQLWN), another plasmid under an arabinose promoter inducible using arabinose (p15a-Pbad-ftsQLWN) (Fig. 3).

**Fig. 5:**
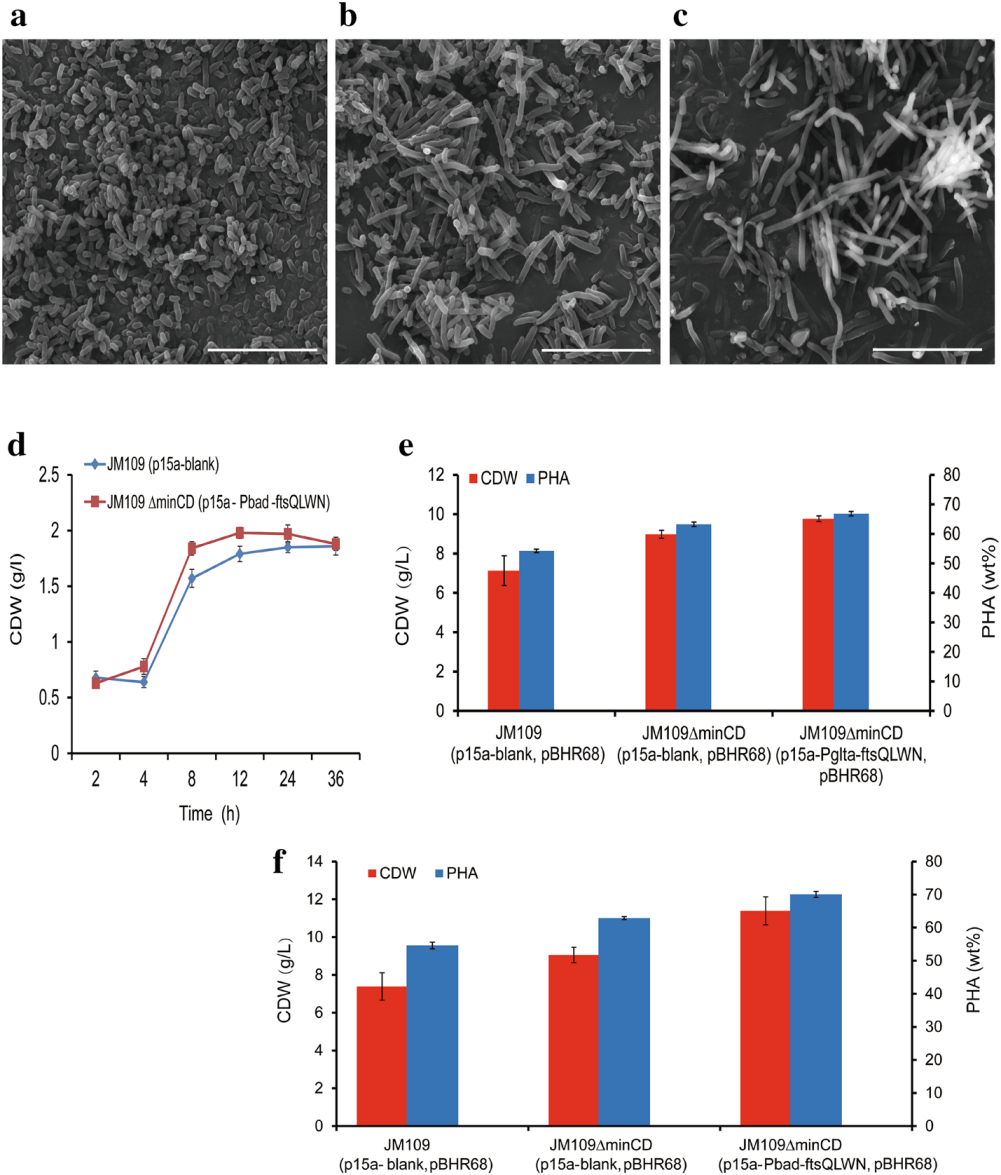
Effect of *ftsQLWN* over-expression on cell morphology and PHA accumulation in *E. coli* JM109 *∆minCD.* SEM morphology studies on: **a** *E. coli* JM109 (p15a-blank). **b** *E. coli* JM109 *∆minCD* (p15a-blank). **c** *E. coli* JM109 *∆minCD* (p15a-Pbad-ftsQLWN). *Scale bars*, 10 μm. **d** The cell growth rate of *E. coli* JM109 *∆minCD* harboring plasmid p15a-Pbad-ftsQLWN.Strains were grown in the LB medium, and 0.2 % arabinose was added into the medium after 2 h of inoculation. *Error bars* are s.d. (n = 3). **e** The cell dry weight and PHA accumulation by *E. coli* JM109 *∆minCD* (p15a-Pglta-ftsQLWN) with pBHR68, Cells were cultivated in LB mediums referred to containing 20 g/L glucose at 37 °C for 48 h. *Error bars* are s.d. (n = 3). **f** The cell growth and PHA production by *E. coli* JM109 *∆minCD* (p15a-Pbad-ftsQLWN) with pBHR68. The strains were grown in the LB medium at 37 °C to an OD600 = 0.4–0.6 (2 h after injection), followed by induction with 0.2 % arabinose for 6 h, then addition of 20 g/l glucose for another 40 h cultivation. *Error bars* are s.d. (n = 3)

Cell morphology and growth rate of the strains were investigated in LB medium. The length of *E. coli* JM109*∆minCD* (p15a-Pbad-ftsQLWN) was longer than the control (Fig. 5a, b, c). The growth rate of *E. coli* JM109*∆minCD* (p15a-Pbad-ftsQLWN) was a little higher than *E. coli* JM109 (p15a-blank) when cultivated in LB medium adding 0.2 % arabinose after 2 h (Fig. 5d). The growth rate between *E. coli* JM109*∆minCD* (p15a-Pglta-ftsQLWN) and *E. coli* JM109 (p15a-blank) was similar (Additional file 2: Figure S1), indicating the arabinose promoter is better under this situation.

*Escherichia coli* JM109Δ*minCD* harboring plasmid p15a-Pbad-ftsQLWN was added with 0.2 % arabinose after 2 h inoculation. And *E. coli* JM109Δ*minCD* with plasmid p15a-Pglta-ftsQLWN was added with 20 g/L glucose without arabinose. After cultured in LB medium for 48 h, *E. coli* JM109Δ*minCD* (p15a-Pglta-ftsQLWN) could reach 9.77 g/L cell dry weights containing 66.83 % PHB which was also higher than the control accumulating less than 60 % PHB (Fig. 5e). On the other hand, *E. coli* JM109Δ*minCD* (p15a-Pbad-ftsQLWN, pBHR68) grew to 11.39 g/L containing 70.06 % PHB, significantly higher than the control group (Fig. 5f). It was therefore concluded that the cell dry weights and PHB contents were improved by overexpressing the division proteins in the multiple fission *E. coli* JM109∆*minCD*, the fast assembly of divisome complex could help the *minCD* mutant to accumulate more PHB granules. Furthermore, the cell dry weights and PHB contents were both higher in the mutant harboring plasmid p15a-Pbad-ftsQLWN than in the plasmid p15a-Pglta-ftsQLWN, indicating that an inducible promoter was better for PHB production.

### *mreB* in *E. coli* JM109*∆minCD* allowed more PHB accumulation in multiple dividing cells

Since the mini-cells formed during the division process waste time and energy reducing space for PHB accumulation, it would be much better if the sizes of the mini-cells can become larger to be a viable cell. It has been known that enzyme complexes responsible for synthesizing cell elongation specific peptidoglycan are organized by the actin homolog MreB [35, 42].

**Fig. 6:**
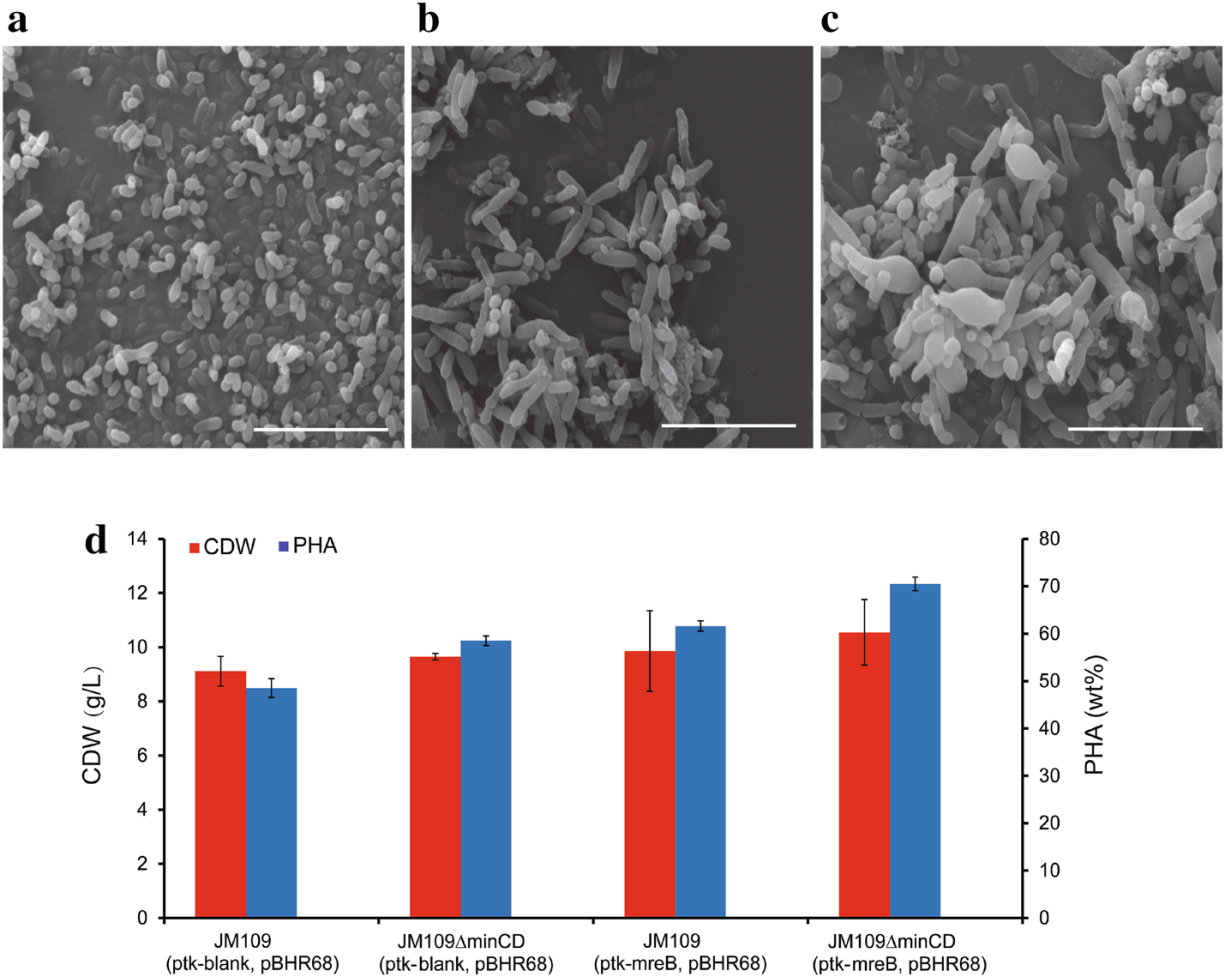
SEM morphology studies and PHA production of *E. coli* JM109 ∆*minCD* overexpressing *mreB*. SEM morphology studies on: **a** *E. coli* JM109 (ptk-blank). **b** *E. coli* JM109 *∆minCD* (ptk-blank). **c** *E. coli* JM109 *∆minCD* (ptk-mreB). *Scale bars*,10 μm. **d** *E. coli* JM109 *∆minCD* overexpressing *mreB* and harboring pBHR68 were cultivated in LB medium, containing 20 g/L glucose at 30 °C for 48 h. *Error bars* are s.d. (n = 3)

*Escherichia coli* changes to spherical shape from rod shape when the expression level of *mreB* changed. By overexpressing *mreB*, sizes of *E. coli* JM109*∆minCD* became larger than that of the control and wild types (Fig. 6a, b, c). When over-expressing *mreB* and PHB synthesis operon *phbCAB* encoded in plasmid pBHR68, *E. coli* JM109*∆minCD* (ptk-mreB, pBHR68) grew to 10.55 g/L containing 70.51 % PHB in the cell dry weights, significantly higher than the wild type grown to 9.11 g/L containing 48.52 % PHB (Fig. 6d). *E. coli* JM109*∆minCD* (ptk-mreB,pBHR68) showed longer and larger sizes (accompanied by many larger mini-cells) compared with the wild type, showing more PHB granule accumulation. Sizes of mini-cells were also improved significantly, permitting more storage of PHB granules.

### Over-expressing genes *ftsQLWN* and *ftsZ* together with scaffold gene *mreB* in *E. coli* JM109*∆minCD* increased cell growth and PHB production

The overexpression of FtsZ could speed up the formation of FtsZ ring, while FtsZ ring related genes *ftsQ, ftsL, ftsW* and *ftsN* (*ftsQLWN*) could accelerate the division process, additional manipulation of *mreB* could enlarge the cell size for more PHB granules accumulation. It was expected that the growth rate of *E. coli* JM109*∆minCD* could be enhanced if all the above genes were functionally expressed.

**Fig. 7:**
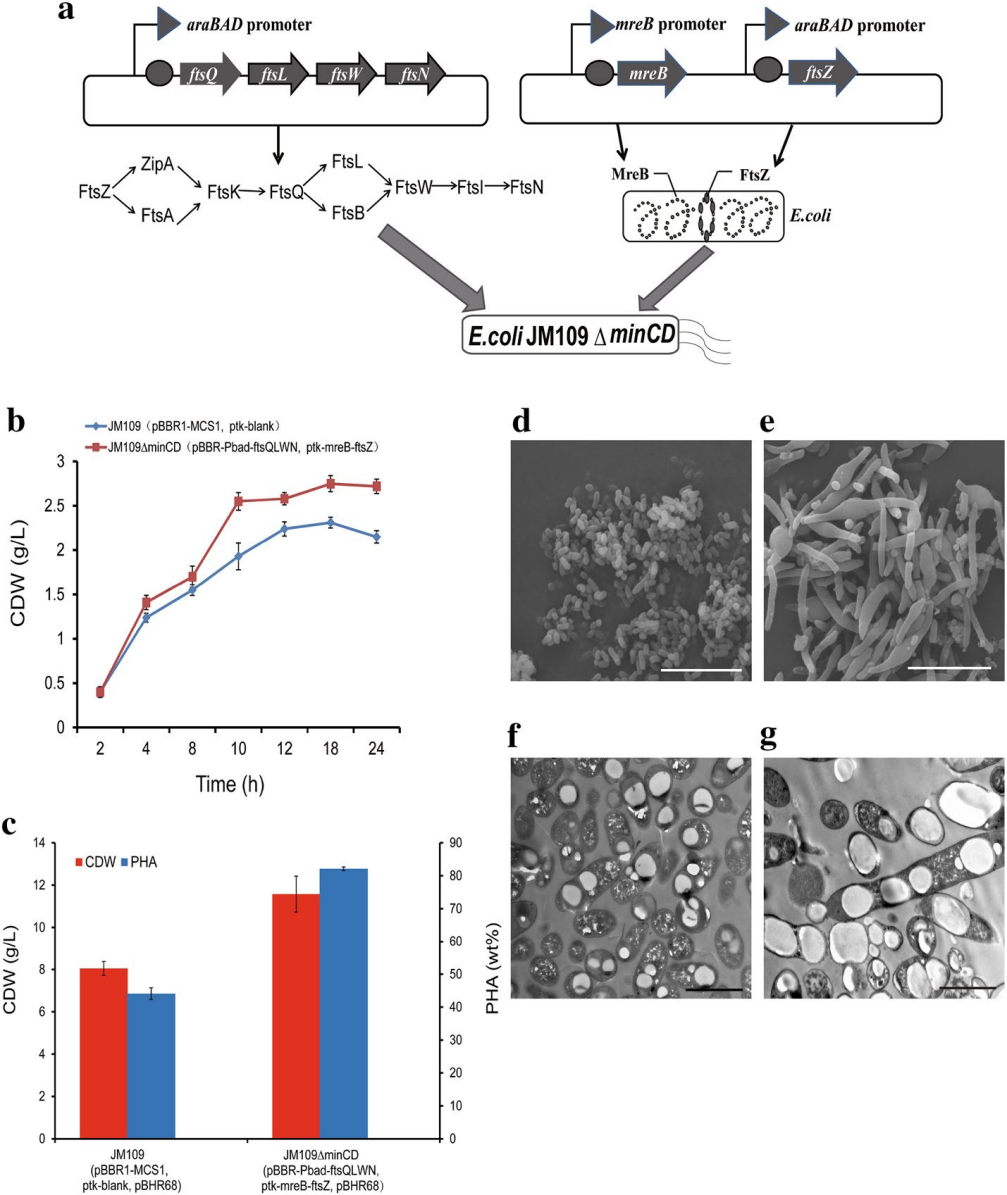
The six genes co-expression system and the influence on cell morphology, PHA production. **a** The co-expression of six genes in *E. coli* JM109 *∆minCD*. The pathway presented in **a** was the order of functional proteins in the division process. FtsA and ZipA bind directly to FtsZ polymers at the division site, followed by the sequential addition of FtsK, FtsQ, FtsL, FtsW, FtsI and FtsN. **b** The cell growth CDW by *E. coli* JM109 *∆minCD* containing plasmid pBBR-Pbad-ftsQLWN and ptk-mreB-ftsZ, the arabinose was added to the culture after 2 h of inoculation. The control was cultivated under the same condition. *Error bars* are s.d. (n = 3). **c** The cell dry weight and PHA contents by the recombinant strain. The strains were grown in the LB medium at 30 °C to an OD600 = 0.4–0.6, followed by induction with 0.2 % arabinose for 6 h, then addition of 20 g/L glucose. *Error bars* are s.d. (n = 3). The SEM results of strains *E. coli* JM109 (pBBR1-MCS1, ptk-blank,pBHR68) (**d**) and *E. coli* JM109 *∆minCD*(pBBR-Pbad-ftsQLWN,ptk-mreB,pBHR68) (**e**), *Scale bars*,10 μm. The TEM of PHB accumulation in *E. coli* JM109 (pBBR1-MCS1, ptk-blank,pBHR68) (**f**) and *E. coli* JM109 *∆minCD* (pBBR-Pbad-ftsQLWN,ptk-mreB,pBHR68) (**g**), *Scale bars*, 2 μm. (**d**–**g**) All the strains were grown in the LB medium at 30 °C to an OD600 = 0.4–0.6, followed by induction with 0.2 % arabinose for 6 h, then addition of 20 g/L glucose for another 40 h of cultivation

Genes *ftsQ*, *ftsL*,*ftsW* and *ftsN* (*ftsQLWN*) were constructed into vector pBBR1-MCS1 to form plasmid pBBR-Pbad-ftsQLWN, which could stably co-exist together with ptk-mreB-ftsZ and pBHR68 in one cell of *E. coli* JM109*∆minCD* (Fig. 7a).

*Escherichia coli* JM109*∆minCD* (pBBR-Pbad-ftsQLWN, ptk-mreB-ftsZ) was then cultivated in LB medium without extra glucose. 20 mL culture broth was sampled regularly during the growth process for CDW. Arabinose was added to the cultures 2 and 4 h after inoculation, respectively. When Z-ring related genes *ftsQLWN* and *ftsZ* were over-expressed at the early stage of growth, the recombinant grew faster to reach 2.8 g/L, higher than 2.2 g/L of the control (Fig. 7b). However, cell growth was not enhanced more when arabinose induction was conducted 4 h after inoculation (Additional file 2: Figure S2). It seemed that gene overexpression in the early stage of cell growth had better effect on cell growth. Thus, arabinose induction was initiated after 2 h of inoculation in subsequent studies.

When over-expressing *ftsQLWN, ftsZ* and *mreB* together in the recombinant strain, *E. coli* JM109*∆minCD* (pBBR-Pbad-ftsQLWN, ptk-mreB-ftsZ, pBHR68) produced 82.13 % PHB in 11.58 g/L CDW, this means a one fold increase in PHB production over the control group (Fig. 7c). It was concluded that overexpression of these six genes together could increase the cell growth rate together with a significant increase on PHB accumulation.

Cell morphologies of recombinants *E. coli* JM109*∆minCD* overexpressing the six genes and PHB synthesis operon were studied under both SEM and TEM (Fig. 7d, e, f, g). It was observed that JM109*∆minCD* (pBBR-Pbad-ftsQLWN, ptk-mreB-ftsZ, pBHR68) was much larger than the control *E. coli* JM109 (pBBR1-MCS1, ptk-blank, pBHR68) (Fig. 7d, e). It was clearly observed that over expression on FtsZ ring and *mreB* genes resulted not only in large cell sizes but also in cells of various morphologies, which could be attributed to the unequal multiple fissions of the cells. The recombinant strain showed more PHB granule accumulation compared with the control group (Fig. 7f, g).

## Discussion

In *E. coli*, the FtsZ ring at mid-cell ensures the binary fission, and the genes encoding proteins MinC and MinD inhibit formation of the FtsZ ring. As a result, overexpression of MinC and MinD in *E. coli* has been known to generate filament cells without FtsZ rings [19]. In contrast, the absence of MinC and/or MinD allows Z rings be formed in any position of a cell either at polar or medial positions [5]. The division pattern of *E. coli* JM109*∆minCD* changed from binary division to multiple fission, and uneven divisions resulted in some larger cells and some normal or smaller size cells. The influence on cell growth rate was limited by *E. coli* JM109*∆minCD*, however, the ability to produce PHB was enhanced, which could be attributed to the long cells generated from the multiple fission possessing more spaces for PHB granule storage, while wild type cells usually have equal small cell sizes.

Since FtsZ ring is important in cell division process, the concentration of FtsZ affects the cell morphology and cell division pattern as reported previously [39]. The length of *E. coli* JM109∆minCD was enhanced further by overexpressing *ftsZ*. PHB accumulation in JM109*∆minCD* (p15a-ftsZ) was elevated and the reason may be attributed to the long shape cell morphology. The high expression level of FtsZ could make cell longer which helped bacterial to store more inclusion bodies, but had little effect on cell growth rate.

In order to enhance the growth rate of *E. coli* JM109*∆minCD*, the division speed should be accelerated. After the formation of FtsZ ring, many genes showed important functions in assembly of the septal ring components, including *ftsQ*, *ftsL*, *ftsW* and *ftsN* [12, 41]. We investigated the function of these four genes together. The cell growth rate had improved a little when overexpress *ftsQ*, *ftsL*, *ftsW* and *ftsN* genes together in *E. coli* JM109∆*minCD*, and the shake flask results demonstrated that the PHB production ability of *E. coli* JM109Δ*minCD* (p15a-Pbad-ftsQLWN, pBHR68) was enhanced significantly. The reason may be attributed to the longer cells with more Z rings of the overexpressing mutant, which could be divided into many daughter cells immediately as soon as the multiple FtsZ rings were formed. Due to the formations of many mini-cells during this multiple fission process, which did not contribute to the cell growth and PHB accumulation as mini-cells had no nucleic acid, the reduction on formations of mini-cells, by overexpression division protein, may be able to improve both cell growth and PHB accumulations.

Since the mini-cells formed during the division process waste time and energy reducing space for PHB accumulation, it would be much better if the sizes of the mini-cells can become larger to be a viable cell. It has been known that enzyme complexes responsible for synthesizing cell elongation specific peptidoglycan are organized by the actin homolog MreB [35]. When overexpressing *mreB* in *E. coli* JM109∆*minCD*, the width of the cell was enhanced and size was bigger compared with wild type, especially within PHB granules. As a result, the ability of PHB production was enhanced by *E. coli* JM109Δ*minCD* overexpressing *mreB*.

As the *ftsZ* and *ftsQLWN* genes could enhance the cell length, and *mreB* gene could enhance the cell width, we constructed two plasmids expressing the six genes together in *E. coli* JM109*∆minCD.* The cell growth rate was accelerated under the function of the high expression level of the six genes. Furthermore, the size of the cell was bigger than the wild type, and the big volume could contain more PHB. The PHB production ability of JM109*∆minCD* (pBBR-Pbad-ftsQLWN, ptk-mreB-ftsZ) was elevated significantly. In conclusion, multiple division and larger cell morphology could enhance the storage space in the cells, and we provide new insights into the PHB production mechanism.

Compared with other efforts to improve PHB production, such as process optimization and PHB pathway engineering et al., the multiple division and larger cell morphology could enhance the production further and provide a new vision on improved PHB production. In order to satisfy the industrialization purpose, there are still many further engineering solutions needed in the new system, such as the improvement on stability of recombinant strain, the reduction of time spending on bacterial division. The work on division pattern and cell morphology of *E. coli* can help improve cell factories more efficiently.

## Conclusions

Bacterial cells are grown in a binary fission way meaning a bacterial cell is equally divided into two (1–2 division). This common growth pattern of *E. coli* can be changed to multiple fission patterns (1 to n, n > 2) by deleting fission related genes *minC* and *minD* together or individually, allowing the formation of multiple fission rings (FtsZ-rings) in several positions of an elongated *E. coli* cell. However, even though a bacterial cell was able to divide into more than two daughter cells, we could not observe significant faster cell growth. To further improve cell growth, some genes related to the divison including *ftsQ*, *ftsW*, *ftsN*, *ftsL* and *ftsZ*, together with the cell shape control gene *mreB*, were all overexpressed in *E. coli* JM109*∆minCD*. Different degrees of cell growth changes were visible. The changing pattern of *E. coli* cell growth resulted in more cell dry weights (CDW) and more than 80 % PHB accumulation increases compared to its binary fission control grown under the same conditions. This study clearly demonstrated that the disrupted min system can change the cell division pattern from binary to multiple fission. Combined over-expression of Z ring synthesis genes *ftsQ*, *ftsW*, *ftsN*, *ftsL* and *ftsZ* together with shape control gene *mreB* led to enhanced PHB accumulation.

## Methods

### Bacterial strains, plasmids and culture conditions

All the microorganisms and plasmids used in this study are listed in Table 2. *E. coli* strains were cultivated in Luria–Bertani (LB) medium or mineral medium (MM). LB medium contains (g/L) 10 tryptone, 5 yeast extract and 10 NaCl. MM medium consists of (g/L): (NH4)_2_SO_4_ 2.0, MgSO_4_ 0.2, Na_2_HPO_4_·12H_2_O 9.65, KH_2_PO_4_ 1.5, trace element solution I 10 ml/L and trace element solution II 1 ml/L. The trace element solution I consists of (mg/L): Fe(III)–NH_4_–citrate 5, CaCl_2_ 2 and HCl 1 M. The trace element solution II contains of (mg/L): ZnSO_4_·7H_2_O 100, MnCl_2_·4H_2_O 30, H_3_BO_3_ 300, CoCl_2_·6H_2_O 200, CuSO_4_·5H_2_O 10, NiCl_2_·6H_2_O 20, NaMoO_4_·2 H_2_O 30 and HCl 0.5 M. When antibiotic selection pressure was required, the medium was supplemented with ampicillin (100 μg/mL), kanamycin (50 μg/mL) or chloramphenicol (30 μg/mL). In order to produce PHA by *E. coli*, 20 g/L glucose was added into the culture medium as a carbon source.

**Table 2:** *Escherichia coli* strains and plasmids used in this study

Names	Descriptions	References
*Strains*		
*E.coli* JM109	*end*A1 *rec*A1 *gyr*A96 *thi*-1 *hsd*R17 (r_k_^−^,m_k_^+^) *rel*A1 *sup*E44 D (*lac*-*pro*AB) [F′*tra*D36 *pro*AB *laq*I^q^ZΔM15]	Invitrogen Inc.
*E.coli* JM109Δ*minC*	carrying *minC* gene knock out for mutagenesis	This study
*E.coli* JM109Δ*minD*	carrying *minD* gene knock out for mutagenesis	This study
*E.coli* JM109Δ*minCD*	carrying *minC* and *minD* genes knock out for mutagenesis	This study
*Plasmids*		
pKD46ReA	λ-Red recombinase expression helper plasmid, oriR101, repA101(ts), Amp^R^	Datsenko and Wanner [45]
pKD13	Template plasmid with Kan^R^ gene and FLP recognition target	Datsenko and Wanner [45]
pCP20	FLP recombinase helper plasmid, ts-rep, Amp^R^, Cm^R^	Datsenko and Wanner [45]
p15a-Pbad-ftsQLWN	p15A ori, arabinose promoter inducible expression of *ftsQ,*-*L,*-*W,*-*N* genes, Cm^R^	This study
p15a-Pglta-ftsQLWN	p15A ori, glta promoter control the expression of *ftsQ,*-*L,*-*W,*-*N* genes, Cm^R^	This study
p15a-ftsZ	p15A ori, arabinose promoter inducible expression of *ftsZ* gene, Cm^R^	This study
p15a-blank	p15A ori, blank vector, Kan^R^	This study
ptk-mreB	pTKRED derivate constitutive expression endogenous *mreB* gene, Kan^R^	Jiang et al. [35]
ptk-mreB-ftsZ	pTKRED derivate constitutive expression endogenous *mreB* gene and *ftsZ* gene, Kan^R^	This study
ptk-blank	pTKRED derivate blank vector, Kan^R^	Jiang et al. [35]
pBBR1-MCS1	Cloning vector, Cm^R^	This study
pBBR-Pbad-ftsQLWN	pBBR1-MCS1with arabinose promoter inducible expression *ftsQ,*-*L,*-*W,*-*N* genes, Cm^R^	This study
pBHR68	A pBluescript II SK-derivative containing phbCAB operon from *Ralstonia eutropha* H16 with native promoter, Amp	Spiekermann et al. [46]

### Construction of plasmids and recombinant strains

Molecular cloning standard procedures including DNA amplification, restriction enzyme digestion and other DNA manipulations were employed for plasmids construction [43]. The kits of DNA purification, plasmids isolation and ligation were purchased from Biomed (Beijing, China) and Thermo (Beijing, China). Primers used in this study were all synthesized by Invitrogen Company (Shanghai, China). In order to construct larger plasmid, the Gibson assembly kit was used in this work (NEB, China) [44].

### Genes *minC* and *minD* knockout in *E. coli*

*Escherichia coli* JM109Δ*minCD* mutant was constructed by one-step disruption on the chromosome [45]. 500 bp homologous upstream and downstream of target gene DNA, and the Kan^R^ gene flanked by FLP recognition target (FRT) sites from plasmid pKD13, were used to form the deletion fragment by PCR amplification. The primers used in this study are listed in Additional file 3: Table S1.

After induction by 0.2 % l-arabinose, *E. coli* JM109 with help plasmid pKD46RecA was maintained ice-cold for 30 min and 10 μl of the deletion fragment was added into the competent cells (Bio-Rad Inc., USA). After PCR verification for screening the positive colony and elimination of the Kan^R^ by plasmid pCP20, the deletions were confirmed by PCR analysis and DNA sequencing.

Finally, DNA sequencing was employed to confirm the gene knockout. At the end, chromosomal *minC* and *minD* genes deleted strain *E. coli* JM109 was obtained.

### Scanning electron microscopy (SEM)

Cells were first fixed with 2.5 % (v/v) glutaraldehyde for more than 4 h, and then washed with 0.1 M phosphate-buffered saline (PBS) (pH 7.3) (3 times, 10 min each). After that, the fixed cells were washed by ethanol in concentration gradient (v/v) of 50, 70, 80, 90 and 100 % in a sequential way, and further dehydrated by tertiary butyl alcohol mixed with ethanol in a ratio of 1:1. The sample was treated with pure tertiary butyl alcohol and used for imaging after lyophilized.

### Ultra-thin-section TEM

After 48 h fermentation, the cells were harvested and re-suspended in 0.1 M phosphate-buffered saline (PBS) (pH 7.3), and then chemically fixed in 2.5 % (v/v) glutaraldehyde. After treatments to prepare ultrathin sections, a H-7650B instrument (Hitachi Ltd, Japan) at 120 kV was used in the electron microscopy study.

### Study on cell growth and PHB production

*Escherichia coli* cells were harvested after 48 h cultivation and washed with distilled water. Cell dry weight (CDW) was measured after lyophilization overnight, and the PHB was analyzed by a gas chromatograph after methanolysis reaction at 100 °C for 4 h. A Spectra System P2000 (Thermo Separation, USA) was used to determine the intracellular PHB contents, while pure PHB (Sigma, American) was used as a standard sample in this study.

## Additional files

10.1186/s12934-016-0531-6 The division process of E. coli JM109∆minCD.
10.1186/s12934-016-0531-6 Growth of *E. coli* JM109∆*minCD* (p15a-pglta-ftsQLWN) and *E. coli* JM109 (p15a-blank) in a LB medium. Error bars are s.d. (n=3). **Figure S2.** Growth of *E. coli* JM109 ∆*minCD* containing plasmid pBBR-Pbad-ftsQLWN and ptk-mreB-ftsZ, respectively. Arabinose was added to the culture after 4 h of inoculation. The control was cultivated under the same condition. Error bars are s.d. (n=3).
10.1186/s12934-016-0531-6 Primers used to knock out *minC* and *minD* genes.

## Abbreviations

PHA: polyhydroxyalkanoates
PHB: poly(3-hydroxybutyrate)
OD_600_: the optical density at 600 nm
CDW: cell dry weight
