# Supplementary material for: Enhanced production of polyhydroxybutyrate by multiple dividing E. coli
Source: Microb Cell Fact. 2016 Jul 27;15:128. doi: 10.1186/s12934-016-0531-6 (PMC4964105; doi:10.1186/s12934-016-0531-6)
Supplement: Supplementary file 2 — 10.1186/s12934-016-0531-6 Growth of E. coli JM109∆minCD (p15a-pglta-ftsQLWN) and E. coli JM109 (p15a-blank) in a LB medium. Error bars are s.d. (n=3). Figure S2. Growth of E. coli JM109 ∆minCD containing plasmid pBBR-Pbad-ftsQLWN and ptk-mreB-ftsZ, respectively. Arabinose was added to the culture after 4 h of inoculation. The control was cultivated under the same condition. Error bars are s.d. (n=3). [file 12934_2016_531_MOESM2_ESM.docx]

Additional File 2:


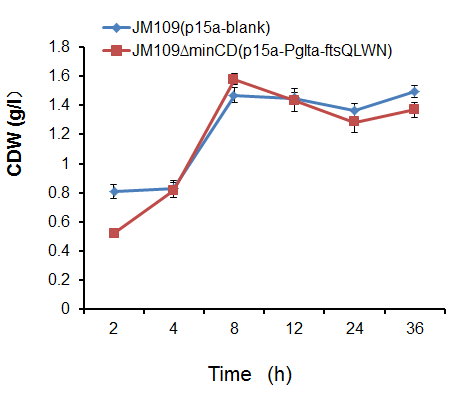


Fig S1 Growth of *E. coli* JM109∆*minCD* (p15a-pglta-ftsQLWN) and *E. coli* JM109 (p15a-blank) in a LB medium. Error bars are s.d. (n=3).


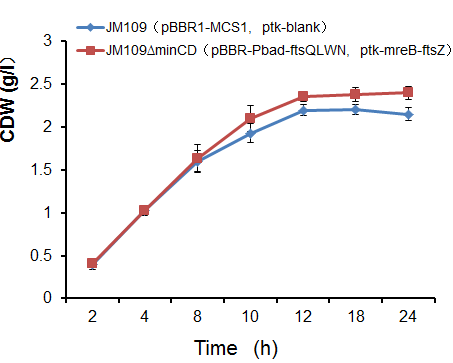


Fig. S2 Growth of *E. coli* JM109 ∆*minCD* containing plasmid pBBR-Pbad-ftsQLWN and ptk-mreB-ftsZ, respectively. Arabinose was added to the culture after 4 h of inoculation. The control was cultivated under the same condition. Error bars are s.d. (n=3).
