# Supplementary material for: Enhanced production of polyhydroxybutyrate by multiple dividing E. coli
Source: Microb Cell Fact. 2016 Jul 27;15:128. doi: 10.1186/s12934-016-0531-6 (PMC4964105; doi:10.1186/s12934-016-0531-6)
Supplement: Supplementary file 3 — 10.1186/s12934-016-0531-6 Primers used to knock out minC and minD genes. [file 12934_2016_531_MOESM3_ESM.docx]

Table S1 Primers used to knock out *minC* and *minD* genes

| Primers | Sequence (5’-3’) |
| --- | --- |
| U-minCD-F | ACCGTACAACACTTATGCTCATTTCATCA |
| U-minCD-R | GGTCGACGGATCCCCGGAATCCTGGCCTTACTCAATTAGCTATTAATCATCG |
| M-minCD-F | CGATGATTAATAGCTAATTGAGTAAGGCCAGGATTCCGGGGATCCGTCGACC |
| M-minCD-R | CGAGAGAAAGAAATCGAGTAATGCCATAACTGTAGGCTGGAGCTGCTTCG |
| D-minCD-F | CGAAGCAGCTCCAGCCTACAGTTATGGCATTACTCGATTTCTTTCTCTCG |
| D-minCD-R | TATCATCTCCCAGTATATCCATACTAACAATAAGGTTATTTACT |
